# Semi-purified extracts of *Commelina benghalensis* (Commelinaceae) induce apoptosis and cell cycle arrest in Jurkat-T cells

**DOI:** 10.1186/1472-6882-14-65

**Published:** 2014-02-20

**Authors:** Kgomotso Welheminah Lebogo, Matlou Phineas Mokgotho, Victor Patrick Bagla, Thabe Moses Matsebatlela, Vusi Mbazima, Leshwene Jeremiah Shai, Leseilane Mampuru

**Affiliations:** 1 Department of Biochemistry, Microbiology and Biotechnology, Faculty of Science and Agriculture, University of Limpopo (Turfloop Campus), Private Bag X1106, Sovenga 0727, Limpopo Province, Republic of South Africa; 2 Department of Biomedical Sciences, Faculty of Science, Tshwane University of Technology, Private Bag X680, Pretoria 0001, Gauteng Province, Republic of South Africa

**Keywords:** Apoptosis, Cell cycle, *Commelina benghalensis*, Jurkat-T cells

## Abstract

**Background:**

*Commelina benghalensis* (CB) is a small plant whose fleshy stems are used in South Africa to treat skin conditions (e.g., cancerous skin outgrowths). This study was aimed at evaluating the effect of sub-fractions of acetone extracts of CB stems on growth-associated molecular events of apoptosis and cell division cycle of Jurkat-T (JT) cells.

**Methods:**

Acetone extract of CB stems were subfractioned into *n*-hexane (F1) and dichloromethane (F2) fractions. After treatment of JT cells with these subfractions, cell proliferation, viability and apoptosis were determined using a haemocytometer, the trypan blue dye exclusion assay, and Hoechst 33258 staining, respectively. Cell division cycle distribution profiles were analysed using an Epics Alba Flow Cytometer and the expression of cell division cycle regulatory genes was analysed using RT-PCR, while immunoreactive proteins were detected on western blots.

**Results:**

The F1 and F2 fractions inhibited the proliferation and viability of JT cells in a concentration- and time-dependent manner, with IC_50_ values of 32.5 μg/mℓ and 56 μg/mℓ, respectively. The observed cytotoxicity was established to be a consequence of apoptosis. as verified using Hoechst staining method. Both fractions induced a G_1_/S interphase arrest of the cell division cycle of JT cells.

RT-PCR analyses showed an up-regulatory effect by the F1 fraction in the expression of *cyclin B1*, *cdc2* and *bax*, with a down-regulatory effect in the expression levels of *bcl-2*. Fraction F1 also increased the protein expression levels of p53 and its downstream regulators, p21 and Cdc2. However, protein Bax and p21 and p53 transcripts were undetectable under the same experimental conditions. On the other hand, fraction F2 increased the mRNA expression levels of *bax*, *bcl-2*, *cyclin B1* and *cdc2*. Concomitantly, fraction F2 showed an up-regulation in the protein expression levels of Cdc2, Bcl-2, Cyclin B1 and p21. Despite the up-regulation in protein expression levels by fraction F2, there was no observable expression levels of the p53 protein and *p21* and *p53* mRNAs under similar experimental conditions.

**Conclusion:**

These findings suggest that the F1 and F2 fractions of CB may provide a valuable lead for the development of novel and effective anti-neoplastic drug(s).

## Background

Cancer is characterised by uncontrolled growth of cells that may arise either due to genetic abnormalities or viral causes. The condition is associated with high morbidity and mortality [1,2]. Although no effective cure, depending on the type of cancer, has been developed, the condition can be managed through the use of radiotherapy and chemotherapy. Indeed, commercially available chemotherapeutics were found to induce death in neoplastic cells [3]. However, these synthetic drugs are mostly non-specific and may be associated with severe side-effects as well as the development of multi-drug resistance phenotypes. In view of the related drawbacks of these chemotherapeutics, the search for new anticancer agents, with a focus on natural compounds from plants, presents an untapped resource base with a huge potential [4]. In fact, plants have been used for the treatment of various disease conditions for centuries in different cultures. With the renewed interest in the use of natural products on a global perspective, formulations derived from natural products with medicinal value may be inexpensive and effective with potential benefit to developing nations [5], especially when these natural products have a history of traditional use.

The main characteristic feature of cancer is unchecked cell growth. As such, understanding the cell division cycle is of primary importance in cancer research. The cell division cycle represents a series of tightly controlled events in which a cell grows in size, duplicates its DNA, segregates its chromatin and divides into two daughter cells [6]. These developmental stages in the cell division cycle have been extensively studied. In Gap phase 1(G_1_), the cell mass increases as the cell prepares to synthesize DNA. This is followed by the synthesis phase (S) where the cell is characterised by DNA synthesis. In Gap phase 2 (G_2_), DNA repair and protein synthesis prepare the cell’s division. The cycle is completed by the mitotic phase (M), in which division of the cell into two daughter cells (cytokinesis) takes place [7]. In some cases, growing cells withdraw from the cell division cycle and enter a resting phase (G_0_) [8,9]. Hence, to ensure accurate replication of the genome and cytokinesis, checkpoints to monitor the integrity of the DNA are placed in the G_1_/S and G_2_/M interphases [10]. These have led to the identification of positive regulators that control cell division cycle [11,12].

When cells are damaged, cell division cycle arrest may occur in either the G_2_/M or G_1_/S interphase to allow complete DNA repair. Extensively damaged cells are committed to undergo apoptotic cell death [13]. Apoptosis is highly regulated and critical for the development and maintenance of healthy tissues. It plays an important role in the protective mechanism against carcinogenesis by eliminating genetically damaged cells, initiated cells or malignant cells. The induction of apoptosis in the treatment of cancer has been considered a highly desirable process, due to its highly ordered nature and complete absence of inflammatory response [14]. Some characteristics of an apoptotic cell are cell shrinkage, chromatin condensation, DNA fragmentation, plasma membrane blebbing, membrane flipping and externalisation of phosphatidyl serine, the formation of apoptotic bodies and activation of caspases [15].

*Commelina benghalensis* L. (CB) is used traditionally as either a food source for its nutritional value or in traditional medicine. The plant is widely used in traditional medicine in China and Nigeria [16,17]. In South Africa, traditional healers use the plant to treat skin outgrowths that are believed to be cancerous. Studies using crude extract of CB from our research group have shown that it possesses anti-neoplastic properties and induces apoptosis in JT cells [18]. In this study, semi-purified extracts of CB were evaluated for their potential growth inhibitory effect and dysregulation of cell division cycle progression of Jurkat-T cells, using standard biochemical and molecular biology techniques.

## Methods

### Preparation of plant material and extraction

*Commelina benghalensis* stems were collected in Bushbuckridge, Mpumalanga Province, South Africa, during summer in dry ice-containing cooler bags. Collected plant material was identified by Prof. J.N. Eloff (University of Pretoria) and voucher specimen number (UL69873) is deposited in the Larry Leach herbarium of the University of Limpopo, Republic of South Africa. The stems were transported within 12 h of harvest and stored at -20°C until required. The frozen stems were minced in liquid nitrogen using a blender and extracted for 24 h with absolute acetone (1 g/10 mℓ). The extracted material was filtered through a Whatman no. 3 filter paper and concentrated using a rotary evaporator (Büchi Labortechnik AG, Switzerland) at 40°C under reduced pressure. The extract residue was then dissolved in ethanol: water (3:1, v/v) and further fractionated with 40 mℓ each of *n*-hexane and dichloromethane to obtain the F1 and F2 fractions, respectively. The fractions were further concentrated with a rotary evaporator at 40°C under reduced pressure. Prior to assaying, the resultant residues were resuspended to an appropriate concentration in 100% dimethylsulfoxide (DMSO).

### Cell culture and treatment

Jurkat-T (JT) cells were obtained from American Type Culture Collection (ATCC, Manassas, VA, USA) and grown in RPMI-1640 media (Adcock-Ingram) supplemented with 10% foetal bovine serum (FBS) (Adcock-Ingram) and 1% antibiotic cocktail (penicillin, streptomycin and neomycin, PSN) (Virbac) in an incubator in a humidified atmosphere of 95% O_2_ and 5% CO_2_ at 37°C. For the treatment of experimental cultures, the stock solutions of the fractions were diluted with RPMI-1640 medium (Adcock-Ingram) supplemented with 10% FBS (Adcock-Ingram) to a final concentration of 0 to 40 μg/mℓ (F1) and 0 to 90 μg/mℓ (F2), respectively. The solutions were filter-sterilised through a 0.22 μm pre-sterilised GP express plus steritop™ filter (Millipore Corporation) before testing. The DMSO concentration in all the treated cultures did not exceed 0.1%. Control cells received an equivalent amount of the highest concentration of DMSO used (0.1%).

### Cell proliferation and viability assays

JT cells were cultured in 6-well tissue culture plates at a density of 1 × 10^5^ cells/mℓ and treated with F1 fraction (0, 10, 32.5 and 40 μg/mℓ) and F2 (0, 30, 56 and 90 μg/mℓ) for 24, 48 and 72 h at 37°C. Cell proliferation was determined using a haemocytometer with the aid of an inverted light microscope (Zeiss, Nikon eclipse Ti). Cell viability was determined by trypan blue dye exclusion assay. Dead and viable cells were counted using a haemocytometer. Viability was expressed as percentage (%) survival per 100 cells counted. DMSO was used as a negative control.

### Morphological evaluation of apoptosis

JT cells were grown in 6-well tissue culture plates at a density of 1 × 10^5^ cells/mℓ and treated with increasing concentrations of the F1 and F2, as above, for 24 h. Cells were then collected by centrifugation at 277 *x g*, washed with 1 × phosphate buffered saline (PBS), pH 7.4, and stained with 20 μg/mℓ Hoechst 33258 for 15 min. The results were observed and recorded under a fluorescence microscope.

### Cell synchronisation and cell cycle analysis

JT cells (8 × 10^5^ cells/mℓ) were arrested at G_0_/G_1_ phase by growing in RPMI-1640 serum-free medium (Adcock-Ingram) for 24 h and released in RPMI-1640 medium (Adcock-Ingram) supplemented with 10% FBS (Adcock-Ingram). The exponentially growing-synchronised JT cells were treated with varying concentrations of F1 fraction (0, 10, 32.5, 40 μg/mℓ) and F2 fraction (0, 30, 56, 90 μg/mℓ) for 24, 48 and 72 h. The cells were then centrifuged at 143 *x g*, fixed with 70% ethanol and stored at -20°C until required.

The fixed cells were washed twice with 1 × ice-cold PBS (pH 7.4) (Adcock-Ingram) containing 1% bovine albumin serum (BSA) (Adcock-Ingram). Cell pellets were resuspended in propidium iodide/RNaseA solution (0.05 mg/mℓ propidium iodide, 40 mM Na^+^-Citrate, pH 7.6, 1 mg/mℓ DNase free-RNaseA, 0.1% NP-40) for 30 min in the dark at 37°C. Cell division cycle distribution profiles were analysed using an Epics Alba Flow Cytometer (Beckman-Coulter, California, USA). Data from 10 000 cells per sample were collected and analysed using the cell Fit Analysis program (Beckman-Coulter).

### Reverse transcription polymerase chain reaction (RT-PCR) analysis

Expression of cell division cycle regulatory genes (*viz*., *bcl-2, bax, p21, cdc2 and cyclin B1*) were analysed using the RT-PCR. Test cultures were treated with F1 and F2 fractions and harvested at 24, 48 and 72 h. Following exposure, cells were washed twice with ice-cold PBS (Adcock-Ingram), pH 7.4, and total RNA isolated using the high pure RNA isolation kit (Roche) according to the manufacturer’s instructions. RNA was quantitated by determining A_260_ readings, with an OD of 1 considered equivalent to 40 μg/mℓ of RNA. The integrity and purity of RNA was evaluated by denaturing formaldehyde gel electrophoresis and by determining the A_260_/A_280_ ratio, respectively. cDNA synthesis was performed using MuLV-RT kit (Perkin-Elmer, Massachusetts, USA) according to manufacturer’s instructions. PCR was performed in a reaction mixture consisting of 4 μℓ cDNA, 1 × PCR buffer with 1.5 mM MgSO_4_, 0.2 μM each primers and dNTPs, and 1.25 units of AmpliTaq DNA polymerase (Perkin–Elmer). The PCR reaction mixture was amplified for 34 cycles on a Hybaid Omnigene thermal cycler, with denaturation at 95°C for 1 min, annealing at 60°C for 1 min (for *bax*, *bcl-2* and *p53*) and 58°C (for *cyclin B1*, *cdc2, p21* and *β-actin*) and extension at 72°C for 1 min and a final extension cycle at 72°C for 7 min. Oligonucleotide primer pairs, synthesised by Invitrogen Life Technologies (UK), were as follows:

***bax****,* 5′-ACCAAAGAAGCTGAGCGAGTGTC-3′ (sense) and 5′-ACAAAGATGGTCACGGTCTGCC-3′ (antisense) [19];

***bcl-2****,* 5′-TGCACCTGACGCCCTTCAC-3′ (sense) and 5′-AGACAGCCAGGAGAAATCAAACAG-3′ (antisense) [19];

***p53****,* 5′-AAAACTTACCAAGGCAACTA-3′ (sense) and 5′-TGAAATATTCTCCATCGAGT-3′ (antisense) [19];

***cyclin B1****,* 5′-AAGAGCTTTAAACTTTGGTCTGGG-3′ (sense) and 5′-CTTTGTAAGTCCTTGATTTACCATG-3′ (antisense) [20];

***cdc-2****,* 5′-GGGGATTCADGAAATTGATCA-3′ (sense) and 5′-TGTCAGAAAGCTACATCTTTC-3′ (antisense) [20];

***p21****,* 5′-CTCAGAGGAGGCGCCATG-3′ (sense) and 5′-GGGCGGATTAGGGCTTCC-3′ (antisense) [20];

***β-actin****,* 5′-GCTCGTCGTCGACAACGGCTC-3′ (sense) and 5′-CAAACATGATCTGGGTCACTTCTC-3′ (antisense) [19].

β-Actin was used as an internal standard. PCR products were analysed on a 1.5% agarose gel containing 0.5 μg/mℓ ethidium bromide, visualised under UV light and photographed using the SynGene Image Analyser (Vacutec, RSA).

### Western blot analysis

After treatment with F1 (0, 30, 56, 90 μg/mℓ) and F2 (0, 10, 32.5, 40 μg/mℓ), JT cells were collected by centrifugation at 277 *× g*, washed twice in 1x ice-cold PBS, pH 7.4, and lysed in 1 mℓ lysis buffer (2 mM Tris–HCl, pH 8.0; 1% Nonidet P-40; 13.7 mM NaCl; 10% glycerol; 1 mM Na_3_VO_4_; 1 mM phenylmethylsulfonyl fluoride; 10 μg/mℓ aprotinin) for 20 min on ice. Lysates were centrifuged at 19 283 *× g* at 4°C for 15 min and aliquots of the supernatants were then used to determine protein concentration using bicinchoninic acid assay (Pierce). Aliquots containing equal amounts of proteins (20-30 μg) were boiled for 3 min in a 2 × sodium dodecyl sulphate (SDS) sample loading buffer [125 mM Tris–HCl, pH 6.8; 4% SDS (w/v); 20% glycerol (v/v); 1 μℓ 2-mercaptoethanol (v/v)] before being resolved on a 12% SDS-polyacrylamide gel (SDS-PAGE). The resolved proteins were electro-blotted onto PVDF-transfer membrane (Millipore Corporation,) using a blotting buffer (10% methanol; 10 mM CAPS, pH 11.0) at 200 mA for 2 h at 4°C. The membranes were blocked with 0.05% TBS-Tween (20 mM Tris–HCl, pH 7.4; 200 mM NaCl) containing 5% non-fat dry milk for 1 h at room temperature. The blocked membranes were washed three times for 10 min with 0.05% TBS-Tween (without milk) and then incubated with specific primary monoclonal/polyclonal antibodies (1:1000) as indicated, *viz*.: goat anti-mouse p21, goat anti-mouse p53, goat anti-mouse Bcl-2, goat anti-mouse Cdc2, goat anti-mouse Cyclin B1, goat anti-mouse β-actin and goat anti-mouse Bax (1:500). The membranes were washed three times with 0.05% TBS-Tween for 10 min and further incubated for 1 h in the presence of a peroxidase-conjugated goat IgG secondary antibody diluted (1:20 000) with blocking buffer. The membranes were washed as described above and immunoreactive proteins detected using the western blotting luminol reagent (Santa–Cruz Biotechnology Inc.) following the manufacturer’s protocol. *β*-Actin was used as the internal control.

### Statistical analysis

The results of each series of experiments, where appropriate, are expressed as the mean values ± standard error of the mean (SEM). Levels of statistical significance were calculated using the paired student *t*-test when comparing two groups or by analysis of variance (ANOVA) with subsequent Tukey-Kramer multiple comparisons test for multiple groups. P values of ≤ 0.05 were considered statistically significant.

## Results and discussions

Developing novel cancer chemotherapeutic agents that have a well-defined mechanism of action is still an emerging field of oncology. In this direction, plants are being thoroughly investigated as a source of novel molecules that can limit cancer growth [3]. Previous studies from our laboratory showed that the crude extract of *C. benghalensis* possesses anti-proliferative effects and induces apoptosis in JT cells [18]. In this study we investigated the effect of semi-purified extracts of *C. benghalensis* on growth-associated molecular events of apoptosis and cell division cycle of JT cells.

### Effects of the F1 and F2 on JT cell proliferation and viability

**Figure 1:**
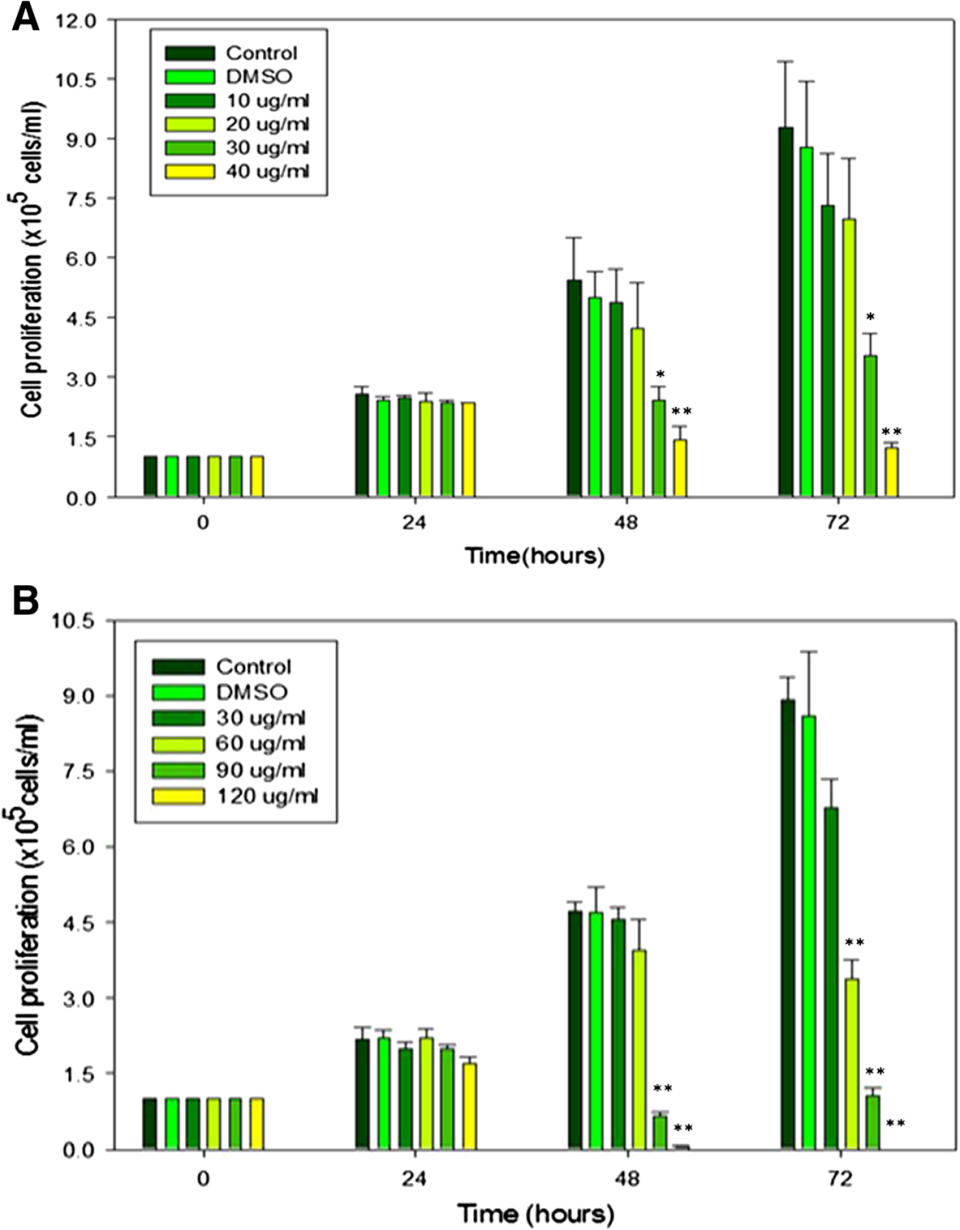
**The anti-proliferative effects of fractions on Jurkat-T cells.** The cells were incubated for 24, 48 and 72 hours with **A**. F1 and **B**. F2 fractions. Control = cells, Negative control =DMSO, *Statistically significant, p≤0.005. **Statistically significant, p≤0.001.

To investigate the effects of the F1 and F2 fractions on cell proliferation, JT cells were treated with different concentrations of both fractions for 24, 48 and 72 h. Both the F1 and F2 fractions inhibited the proliferation of cells in a time- and concentration-dependent manner (Figures 1A and B). Cells were incubated for 24, 48 and 72 h in the presence or absence of different concentrations of the F1 and F2 fractions and the cell numbers were determined using a haemocytometer. The results are presented as the mean ± SEM of two independent experiments, each performed in duplicate. The final concentration of DMSO used in all the treated cells was less than 0.1%.

**Figure 2:**
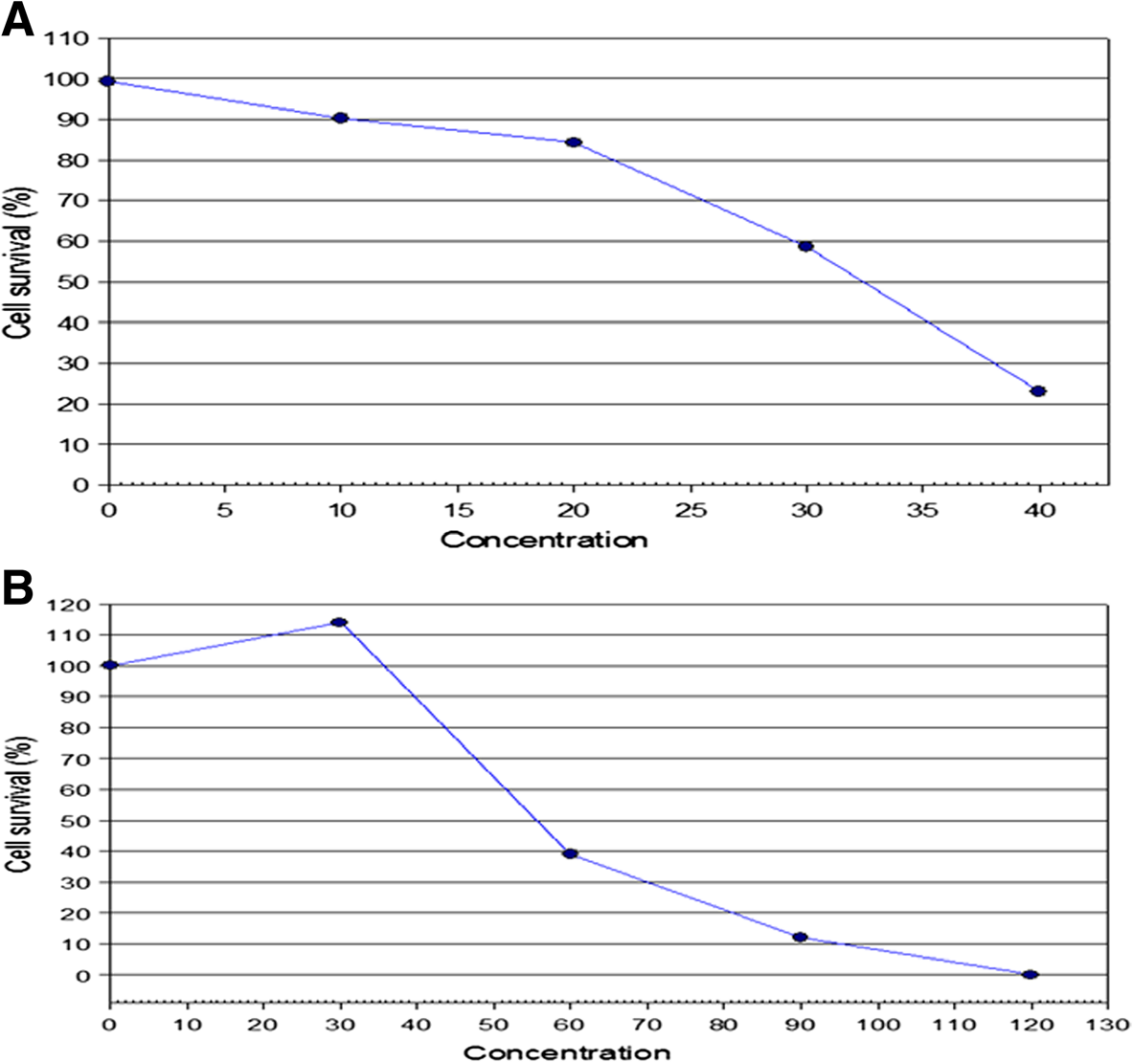
**IC**_**50 **_**value of the treated Jurkat-T cells.** The cells were incubated for 24, 48 and 72 hours in the presence and absence of different concentrations of the **A**. F1 and **B**. F2 fractions.

**Figure 3:**
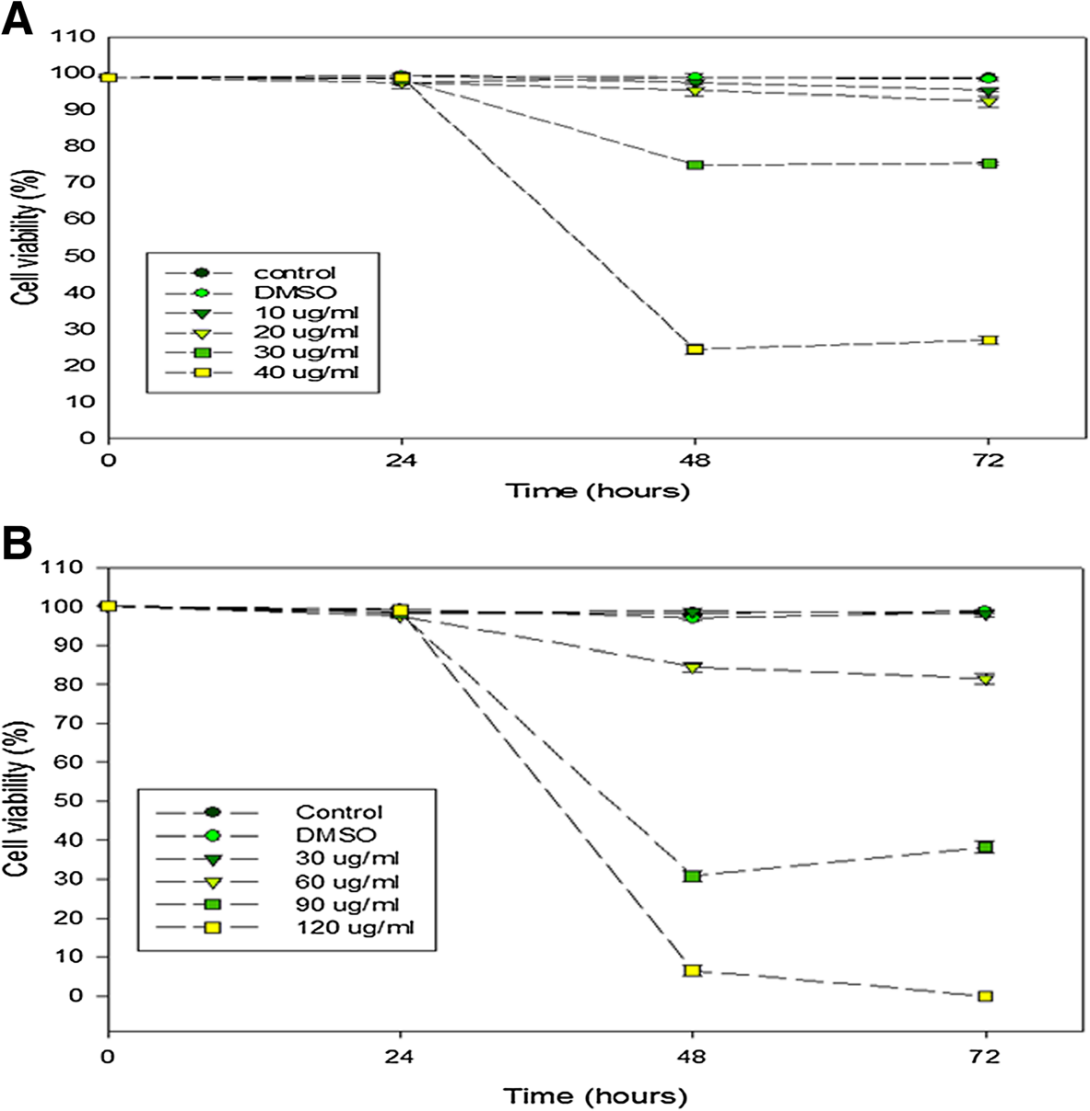
**Effects of the fractions on the viability of Jurkat-T cells.** The cells were incubated for 24, 48 and 72 hours in the presence and absence of different concentrations of the **A**. F1 and **B**. F2 fractions.

At 24 h of incubation with the F1 fraction, a marked variation was not observed between the various concentrations used, even at the highest concentration of 40 μg/mℓ. With prolonged extended time of incubation, an increase in cell proliferation was observed at lower concentrations with a concomitant anti-proliferative effect at the highest concentrations of 30 - 40 μg/mℓ. A similar trend was observed with the F2 fraction, suggesting that the anti-proliferative effect of both fractions was evident only at higher concentrations with extended time of exposure. A measure of the antagonist drug potency (IC_50_) in both fractions was determined (Figures 2A and B). Given that the different concentrations of the two fractions were used in the study, the trend in cell viability decreased with increase in the concentration of the F1 and F2 fractions (Figures 3A and B).

Both F1 and F2 fractions displayed potent anti-proliferative activity, with IC_50_ for F1 fraction at 32.5 μg/mℓ, and 56 μg/mℓ for F2 fraction. The Monkey Vero cells were used as normal cells to determine the cytotoxicity of both fractions used. Concentrations of up to 250 μg/mℓ of either fraction had no inhibitory effect on these cells (data not shown). The strong growth inhibitory activity of both fractions prompted us to determine their possible mode of molecular mechanism(s) of action. Since JT cells are irreversibly transformed, they have a way of evading apoptosis. Hence, the main target of any putative and novel bioactive compound isolated from medicinal plants would be to trigger apoptosis in these neoplastic cells, while inhibiting their proliferation.

### F1 and F2 fractions induce apoptotic cell death in JT cells

**Figure 4:**
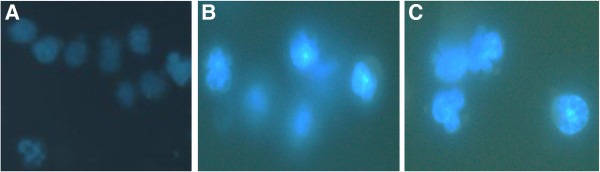
**Inhibition of cell cycle progression in Jurkat-T cells after treatment.** The cells were incubated with or without the F1 fraction for 24, 48 and 72 hours. Cells were fixed with 70% ethanol, stained with propidium iodide and then the cell cycle distribution was analyzed. The cells were released from synchronization prior to treatment with the **A**. F1 fraction. A = serum starved cells for 24 hours, B = control cells, C = cells treated with F1 fraction at 10 μg/mℓ, D = cells treated with F1 fraction at 32.5 μg/mℓ, E = cells treated with F1 fraction at 40 μg/mℓ. **B**. F2 fraction. A = serum starved cells for 24 hours, B = control cells, C = cells treated with F2 fraction at 30 μg/mℓ, D = cells treated with F2 fraction at 56 μg/mℓ, E = cells treated with F2 fraction at 90 μg/mℓ. Note the distinct accumulation of cells in the S phase. These results are representative profiles of the cell division cycle at 72 hours.

To further investigate whether the death of the JT cells was due to the process of apoptosis, and not necrosis, the morphological changes indicative of the apoptotic process were assessed. Morphological changes in the nuclear chromatin during apoptosis can be detected by Hoechst 33258 staining. In this assay, apoptotic cells are identified by bright blue nucleus as the chromatin is condensed, while nuclei of non-apoptotic cells stain a weak and homogenous blue colour due to the evenly spread and monogranulated chromatin. After treating the cells with F1 and F2 fractions for 24 h, the blue emission light, indicative of apoptosis, in apoptotic cells was brighter than in the control cells. Condensed chromatin was also observed in a large number of fractions F1- and F2-treated cells with the presence of apoptotic bodies evident in some cells (Figure 4).

### Effects of F1 and F2 fractions on cell division cycle progression of JT cells

**Figure 5:**
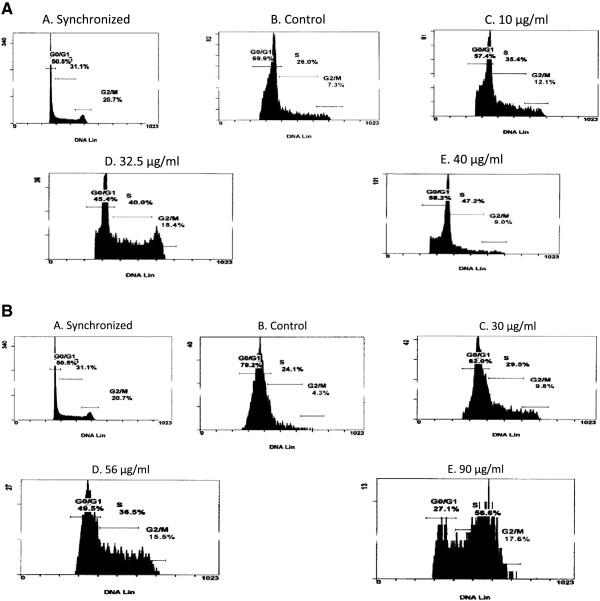
**Effects of the fractions on mRNA expression of bax, bcl-2, cdc2 and cyclin B1 in Jurkat-T cells. A**. F1 fraction; lane 1 = control; lane 2 = 10 μg/ml; lane 3 = 32.5 μg/mℓ and lane 4 = 40 μg/mℓ. **B**. F2 fraction; lane 1 = control; lane 2 = 30 μg/mℓ; lane 3 = 56 μg/mℓ and lane 4 = 90 μg/mℓ. Total RNA was prepared and RT-PCR performed as described in the materials and methods. β-actin was used as the internal control.

The effects of F1 and F2 fractions on the cell division cycle distribution profiles were assessed by flow cytometry. After 24, 48 and 72 h of treatment, 40 μg/mℓ of fraction F1 was observed to gradually increase the S phase population (Table 1 and Figure 5A) from 29.28% at 24 h to 41.53% at 72 h, suggesting an increase in S phase population with prolonged exposure. This increase was accompanied by a concomitant decrease in the number of cells in the G_0_/G_1_ phase (66.73% at 24 h, 63.33% at 48 h, 41.75% at 72 h) with prolonged exposure time.

**Table 1:** Cell cycle distribution profiles of Jurkat-T cells treated with different concentrations of the F1 fraction

**Cell cycle phase**	**Time (hours)**	**0 μg/mℓ**	**10 μg/mℓ**	**32.5 μg/mℓ**	**40 μg/mℓ**
**G**_**0**_**/G**_**1**_	24	65.95% ± 6.70	63.95% ± 1.55	68.65% ± 6.00	66.73% ± 2.08
	48	64.50% ± 3.46	62.25% ± 0.05	62.00% ± 0.25	63.33% ± 0.68
	72	58.00% ± 1.31	60.03% ± 1.63	51.88% ± 8.43	41.75% ± 2.03
**S**	24	28.73% ± 5.42	31.20% ± 0.65	28.33% ± 3.83	29.28% ± 0.48
	48	28.28% ± 4.38	33.58% ± 2.03	32.30% ± 3.65	27.65% ± 2.30
	72	38.53% ± 4.58	36.08% ± 0.43	38.70% ± 2.35	41.53% ± 3.08
**G**_**2**_**/M**	24	7.28% ± 1.23	7.73% ± 0.28	7.48% ± 0.28	5.88% ± 3.33
	48	11.00% ±1.18	9.03% ± 2.98	9.13% ± 2.98	12.28% ± 0.83
	72	9.08% ± 3.88	6.30% ± 5.58	13.68% ± 5.58	4.95% ± 0.00

Similarly, fraction F2 was also observed to arrest the cell division cycle progression at the S phase (Figure 5B). Fraction F2, at 90 μg/mℓ, showed a pronounced increased in the S phase population from 29.35% at 24 h to 47.80% at 72 h (Table 2). This was accompanied by a sharp decline of G_0_/G_1_ phase cell population from 62.03% at 24 h, 58.50% at 48 h and 44.35% at 72 h (Table 2 and Figure 5B), suggesting that both fractions F1 and F2 alter the cell division cycle by arresting JT cells in the S phase.

**Table 2:** Cell cycle distribution profiles of Jurkat-T cells treated with different concentrations of the F2 fraction

**Cell cycle phase**	**Time (hours)**	**0 μg/mℓ**	**30 μg/mℓ**	**56 μg/mℓ**	**90 μg/mℓ**
**G**_**0**_**/G**_**1**_	24	69.68% ± 1.18	69.35% ± 2.10	67.13% ± 3.88	62.03% ± 0.93
	48	61.13% ± 4.23	61.08% ± 1.28	51.65% ± 0.8	58.50% ± 2.95
	72	64.83% ± 11.23	61.30% ± 3.80	55.30% ± 0.2	44.35% ± 7.80
**S**	24	23.63% ± 0.93	26.28% ± 2.28	24.45% ± 3.45	29.35% ± 3.25
	48	29.15% ± 2.70	29.53% ± 1.18	37.68% ± 0.88	29.88% ± 0.18
	72	29.23% ± 5.68	36.90% ± 7.95	39.45% ± 5.5	47.80% ± 0.50
**G**_**2**_**/M**	24	9.08% ± 0.63	8.03% ± 1.68	12.40% ± 2.05	11.40% ± 1.80
	48	12.00% ± 1.45	11.35% ± 2.3	12.50% ± 0.15	13.93% ± 3.43
	72	6.65% ± 0.00	5.50% ± 0.00	6.65% ± 0.00	11.85% ± 5.10

### Effects of the F1 and F2 fractions on the mRNA expression levels of *p21*, *p53*, *cyclin B1*, and *cdc2*

**Figure 6:**
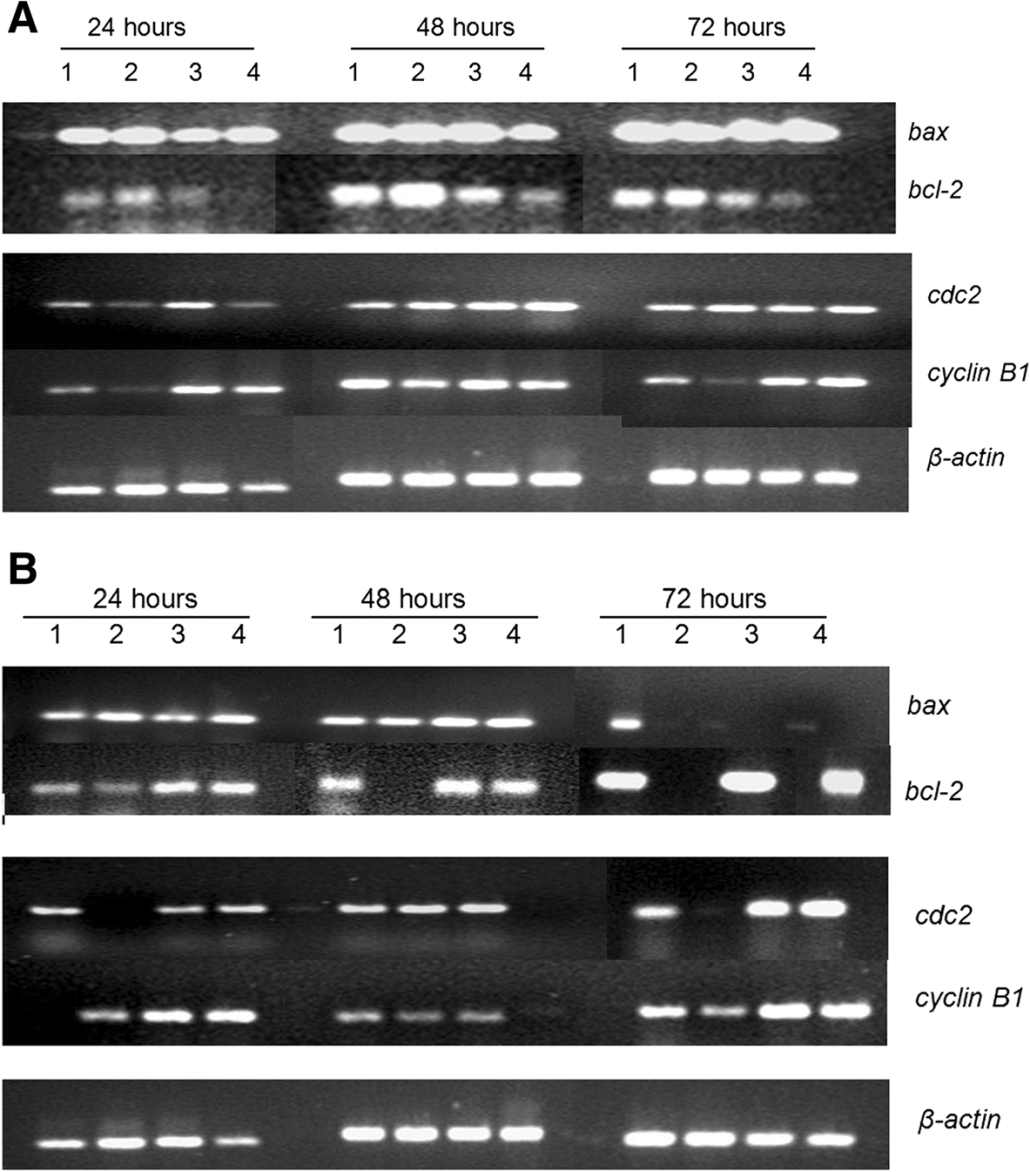
**Effects of the F1 fraction on the expression levels of p53, p21, Bcl-2 and Cdc2 in Jurkat-T cells.** Lane 1 = control; lane 2 = 10 μg/mℓ; lane 3 = 32.5 μg/mℓ; lane 4 = 40 μg/mℓ. Total cellular proteins were prepared and western blot performed as described in the materials and methods. Note the kinetics of p21 up-regulation which coincided with the up-regulation of p53. β-actin was used as the internal control.

The progression of the cell division cycle is largely controlled by cyclins, the regulatory units of CDKs, and CDKIs. The expression levels of *cyclin B1* mRNA increased following fraction F1 treatment at 32.5 μg/mℓ and 40 μg/mℓ after 24 and 72 h. The mRNA expression levels of its catalytic partner, *cdc2*, were also increased after 48 h following fraction F1 treatment under the same treatment conditions (Figure 6A). On the other hand, treatment of JT cells with fraction F2 also resulted in an increase in the expression levels of *cyclin B1* mRNA at 24 and 72 h, while *cdc2* mRNA expression levels were only up-regulated at 72 h (Figure 6B), suggesting a time lag between the two fractions in the up-regulation of *cdc2* mRNA expression levels. Although the initial events promoted by F1 fraction on JT cells before the commencement of apoptosis are not well understood, it may be likely that the F1 fraction may have increased the expression of certain genes that may subsequently be important in mediating the early-response initiation of apoptosis, which was not the case with the F2 fraction. However, *p53* mRNA and its downstream regulator, *p21* mRNA, for both F1 and F2-treated JT cells did not display any detectable levels possibly due to their unstable nature and short half-lives (data not shown).

Indeed, mRNA degradation is said to be a tightly regulated process dependent on specific *cis*- and *trans*-acting factor interactions [21]. Many mRNAs that are subjected to message turnover bear AU-rich elements (AREs) in their 3′-untranslated regions (3′ UTRs), which often contain the pentamer AUUUA [22]. To investigate why fractions F1- and F2-treated cells did not show any detectable levels of both *p21* and *p53* mRNAs in the RT-PCR analysis, the time points in a subsequent experiment were adjusted (*i.e.*, 6, 12, 24 and 48 h). Despite these adjustments, we could still not detect any appreciable expression levels of *p21* and *p53* mRNAs. This finding may suggest that the mRNA transcripts for both genes may have been possibly degraded.

### Effects of the F1 and F2 fractions on the mRNA expression levels of *bax* and *bcl-2*

In the group of genes associated with apoptosis, the *bcl-2* family is one of the most important groups due to its regulatory role during apoptosis. Among this family of genes, *bcl-2* is anti-apoptotic, whereas *bax* is known as the pro-apoptotic gene. The treatment of the JT cells with F1 fraction resulted in a decrease in the *bcl-2* mRNA expression levels in a time- and concentration-dependent manner, followed by a simultaneous increase in the *bax* mRNA expression levels (Figure 6A). The decrease in the *bcl-2* expression levels was more prominent at 48 and 72 h after treatment with 32.5 μg/mℓ and 40 μg/mℓ, whereas the increase in the *bax* expression levels was observed at all-time points tested (24-72 h) (Figure 6A). In view of the fact that *bax* genes code for proteins that affect the susceptibility of cells to undergo apoptosis, it may be likely that the amount of Bcl-2 and Bax proteins in JT cell vary coordinately or that the effect of F1 fraction may have influenced a drop in Bcl-2 levels before the onset of apoptosis.

The expression levels of *bcl-2* mRNA and *bax* mRNA in JT cells treated with F2 fraction were also investigated. Expression of *bcl-2* mRNA slightly increased at 24 h at 56 μg/mℓ and 90 μg/mℓ (Figure 6B). The expression of *bax* mRNA was up-regulation at 48 h after exposure to F2 fraction at 56 μg/mℓ and 90 μg/mℓ. At 72 h, however, fraction F2 treatment decreased *bax* mRNA expression.

### Effects of F1 and F2 fractions on the protein levels of Bax and Bcl-2

**Figure 7:**
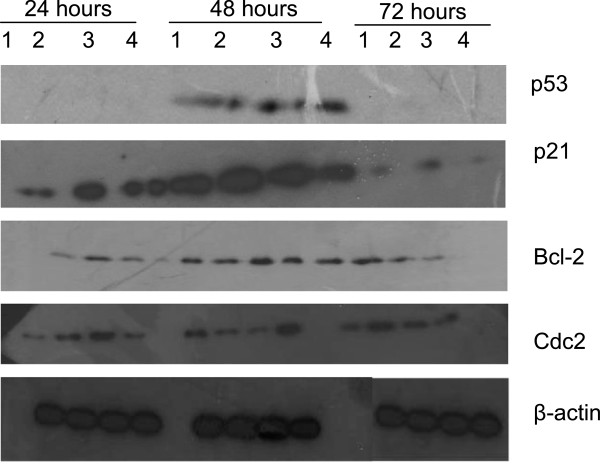
**Morphological changes of the nuclei of treated Jurkat-T cells.** Cells were treated with 0.09% DMSO **(A)**, 40 μg/mℓ of F1 **(B)** and 90 μg/mℓ of F2 **(C)** fraction and stained with Hoechst 33258 stain after 24 hours. Note the presence of apoptotic features such as nuclear shrinkage and chromatin condensation are prominent in **B** and **C**. The experiment was repeated twice to ascertain reproducibility.

Western blot analyses of JT cells demonstrated a down-modulation of the expression level of Bcl-2 in the presence of F1 fraction at 32.5 μg/mℓ and 40 μg/mℓ after 72 h (Figure 7). The expression levels of *bcl-2* mRNA in fraction F2-treated JT cells were significantly down-modulated at all the time points tested (24-72 h) (Figure 6B). On the other hand, Western blot analysis of Bax protein of fractions F1- and F2-treated JT cells did not display any detectable levels (data not shown). The observed phenomenon may likely be due to the fact that the Bax protein may have been subjected to proteasome-mediated degradation under the experimental conditions used.

The western blot analysis of p53 in the fraction F2-treated JT cells did not exhibit any detectable levels. The p53 protein has been found to have a short half-life due to the intricate regulation processes that it has to undergo, including reversible cycles of post-translational modification such as phosphorylation, acetylation and ubiquitination [23]. Under normal conditions, the p53 tumour suppressor protein has been considered to be rapidly degraded by the 26S proteasome in a process mediated by the Mdm2 protein and Jun kinase [24]. Since this study did not demonstrate any detectable levels of p53, at all the time points tested, the possibility that the p53 protein could have undergone proteasomal degradation cannot be ruled out. However, despite the fact that p53 levels were not detected under the current experimental conditions, p21Waf1/Cip1 protein levels were highly up-regulated and cell cycle arrest was induced in the G_1_/S interphase and with cells eventually undergoing apoptosis. p21Waf1/Cip1, in addition to induction of cell cycle arrest in the G_1_/S interphase, can induce apoptosis in these cells. The induction of apoptosis by p21Waf1/Cip1 was not found to be extensive as compared to that induced by p53, but it was nonetheless still detectable. These results suggested that p21Waf1/Cip1 induces apoptosis in a p53-independent manner in the fraction F2-treated JT cells. This also suggests that the up-regulation of p21Waf1/Cip1 may be related to fraction F2-induced G_1_/S interphase block and growth inhibition in JT-cells.

Since our results demonstrated an up-regulation in the protein expression levels of p21Waf1/Cip1, which led to the arrest of cell cycle in the G_1_/S interphase, it was essential to investigate its effects on the cell division cycle regulatory proteins operative within the G_1_/S interphase, including Cyclin B1 and Cdc2. The products of these cell division cycle regulatory genes are critical determinants of the progression of cancer. Their dysregulation is considered to have an adverse effect on the normal cycling of cells [25].

Consequently, RT-PCR analysis of fraction F1-treated JT cells revealed an up-regulation in the expression levels of *cyclin B1* mRNA at 24 and 72 h and an up-regulation of its catalytic partner, *cdc2* mRNA, after 48 h at concentrations of 56 and 90 μg/mℓ. Western blot analysis of the fraction F1-treated JT cells demonstrated an up-regulation in the expression levels of the Cdc2 protein after 48 h at concentrations of 56 and 90 μg/mℓ. Further, RT-PCR analysis of the fraction F2-treated JT cells resulted in an up-regulation in the expression levels of *cyclin B1* mRNA at 24 and 72 h, whereas the expression levels of its catalytic partner, *cdc2*, were up-regulated after 72 h. Contrary to the *cdc2* mRNA RT-PCR data, the Cdc2 protein was up-regulated at all the time points tested (24, 48 and 72 h). Indeed, an up-regulation in the expression levels of the Cdc2 protein is considered to be a common feature among apoptosis induced by any form of stimuli without an effect on the cell division cycle progression [26]. Our findings, therefore, suggest that the up-regulation in the expression levels of Cdc2 protein after treatment with fractions F1 and F2 could have possibly led to the induction of apoptosis independent of the G_1_/S interphase arrest.

Western blot analysis of Cyclin B1 of the fractions F1- and F2-treated cells also did not show any detectable levels. This could have possibly been due to the fact that Cyclin B1 protein may have been subjected to a proteasomal degradation process. This is so because intracellular protein degradation plays an essential role in many physiological processes by removing damaged proteins that harbour destruction (ubiquitin) tags. In eukaryotic cells, degradation of cytosolic and nuclear proteins is mainly mediated by the 26S proteasome [27].

The key biochemical events involved in the induction of apoptosis are the up-regulation of pro-apoptotic proteins and/or down-regulation of anti-apoptotic proteins. Therefore, the ratio of pro-apoptotic to anti-apoptotic proteins of the Bcl-2 family is an important determinant of a cell’s bias to undergo apoptosis. The Bcl-2 family of proteins either promotes cell survival (e.g., Bcl-2) or induces apoptosis (e.g., Bax). Increased levels of Bax and concomitant decreased levels of Bcl-2 have been suggested to permeabilise the mitochondria to release pro-apoptotic factors such as cytochrome C, which is upstream of caspase-3 activation [13]. Subsequently, the present study demonstrated a time- and concentration-dependent decrease in the expression levels of *bcl-2* mRNA and increased levels of *bax* mRNA in the fraction F1-treated JT cells. On the other hand, there was a down-modulation of the Bcl-2 protein after 72 h treatment with F2 fraction, at concentration of 32.5 and 40 μg/mℓ. Furthermore, treatment of cells with fraction F2 resulted in an up-regulation in the expression levels of *bax* mRNA at 48 h and *bcl-2* mRNA after 24 h, both at concentrations of 56 and 90 μg/mℓ.

Since biological processes are normally driven by proteins, immunoblot analysis was done in order to confirm the RT-PCR results. The Western blot results for fraction F2-treated JT cells did not corroborate the observations made from the RT-PCR data. There was a time- and concentration-dependent decrease in the expression levels of Bcl-2 protein in fraction F2-treated cells. Several reasons pertaining to this discrepancy can be suggested. This inconsistency could be a consequence of differences in post-translational regulatory mechanisms, as well as differences in mRNA and protein turnover [28].

Western blot analysis of the Bax protein, for fractions F1- and F2-treated cells, did not show any detectable levels. It has been shown previously that certain apoptosis-promoting proteins such as Bax are subjected to proteasome-mediated degradation [29]. It also has been suggested that Bax, as compared to Bcl-2, is a direct target protein of the ubiquitin/proteasome pathway [30]. p53 induces apoptosis through the induction of Bax. Thus, the ubiquitin-proteasome may negatively regulate p53-dependent apoptosis by degrading the p53 downstream target, the Bax protein [23].

The time points that were used for the Western blot analyses (24, 48 and 72 h) seemed not to have been ideal for the detection of the Bax protein in fractions F1- and F2-treated JT cells. This observation may suggest that the Bax protein messages could have been translated within 24 h and as such could not be possibly detected beyond this time point. Consequently, another experiment with earlier sampling time-point intervals (6 and 12 h) was conducted, but could still not detect any expression levels of Bax protein. Therefore, we speculate that the Bax protein could have undergone ubiquitin proteasomal degradation. Despite the non-detection of the Bax protein, the treated cells did undergo apoptosis as evidenced by the down-regulation of the anti-apoptotic protein Bcl-2. This finding could, indeed, explain the disproportionate expression profiles of both p53 and Bax proteins observed in the present study.

The above information could buttress the idea that JT cells, under the conditions of fraction F1 and F2 treatment, undergo apoptosis independent of the Bax protein. It also shows that p53 repressed the expression of Bcl-2 in the fraction F1-treated JT cells and thereby contributed to apoptosis by blocking survival signals mediated by Bcl-2. The data also suggested that the down-modulation of Bcl-2 in fraction F2-treated JT cells might be another molecular mechanism through which fraction F2 induces apoptosis; however, this explanation needs to be explored.

Our findings also suggest that fraction F1 inflicted an insult to the JT cells, thus leading to the activation of the tumour suppressor protein, p53. p53 in turn up-regulated the expression levels of p21Waf1/Cip1, thus leading to the arrest of cells in the G_1_/S interphase. The resultant G_1_/S interphase arrest meant that the cells were given enough time for DNA repair. However, since the damage was irreparable, the p53 promoted apoptosis by down-regulating the expression levels of Bcl-2 and the damaged JT cells were then eliminated. In the case of the fraction F2-treated JT cells, we suggest that p21waf1/Cip1 played a dual role of arresting and inducing apoptosis in the JT cells. Fraction F2-treated JT cells also induced cell cycle arrest in the G_1_/S interphase, with cells consequently undergoing apoptosis through the down-modulation of the expression levels of Bcl-2 protein.

## Conclusion

Results from the current study showed that the F1 and F2 fractions exhibit remarkable growth inhibitory effects against JT cells. The study also demonstrated that JT cells treated with different concentrations of F1 and F2 fractions display some of the classical morphological hallmark features of apoptosis, such as cell shrinkage, chromatin condensation, DNA fragmentation and plasma membrane blebbing. Fraction F1-treated JT cells also demonstrated increased expression levels of p21Waf1/Cip1 and p53 and a simultaneous arrest of the cell division cycle in the G_1_/S interphase. This finding suggests that the induction of p21Waf1/Cip1 is p53-dependent and that p21Waf1/Cip1 plays a role in fraction F1-induced G_1_/S arrest of treated JT cells.

Fraction F1 is also shown to inhibit cell proliferation accompanied by the up-regulation of p53, p21Waf1/Cip1 and Cdc2, while fraction F2 inhibits cell proliferation via the up-regulation of p21Waf1/Cip1 and Cdc2. It is thus likely that both fractions induce apoptosis through a down-regulation of Bcl-2 in JT cells. However more studies are required to buttress this assumption. Hence, fractions F1 and F2 of CB may have anti-tumour effects which are mediated through the G_1_/S interphase arrest. Furthermore, these results suggest that CB may have a potential to be developed as a therapeutic agent against cancer. Although the study presents preliminary data, the results somewhat validate the ethnopharmacological usage of this plant in the treatment of malignant phenotypes. Future studies will focus on the understanding of the actual mechanism(s) of action that is (are) associated with the observed cell death events. Chemical structure elucidation of the bioactive compounds, that are present in and are responsible for the observed activity elicited by fractions F1 and F2, is also pertinent**.**

## Competing interests

The authors declare to have no competing interests.

## Authors’ contributions

KWL, MPM and VM carried out the experiments and analysed the data. TMM and LJM made substantial contribution to the conception and design of the study. VPB and LJS revised the manuscript critically. All authors read and approved the final manuscript.

## Pre-publication history

The pre-publication history for this paper can be accessed here:

http://www.biomedcentral.com/1472-6882/14/65/prepub
